# Bridging innate and adaptive tumor immunity: cGAS–STING pathway activation to potentiate immune checkpoint blockade

**DOI:** 10.1186/s13046-025-03555-9

**Published:** 2025-11-07

**Authors:** Zhuo Li, Wei Zheng, Yisi Liu, Rong Cao, Jing Wei, Haiqing Jia

**Affiliations:** Department of Gynecology, Cancer Hospital of Dalian University of Technology, Liaoning Cancer Hospital & Institute, Shenyang, 110042 China

**Keywords:** CGAS–STING pathway, STING agonist, Immune checkpoint inhibitor, Tumor microenvironment, Cancer immunotherapy

## Abstract

The cyclic GMP–AMP synthase–stimulator of interferon genes (cGAS–STING) pathway is critical for innate immunity, as it detects cytoplasmic DNA and drives type I interferon signaling. Pharmacological stimulation of this pathway has been recognized as a valuable approach for cancer immunotherapy, especially when used together with immune checkpoint inhibitors (ICIs). Preclinical studies have demonstrated synergistic antitumor effects of cGAS–STING agonists and ICIs across various tumor models, while early-phase clinical trials are exploring their safety and efficacy in patients. Nonetheless, intrinsic tumor resistance, an immunosuppressive tumor microenvironment (TME), and therapy-associated immune toxicities continue to pose substantial obstacles to clinical application. In this review, we provide an overview of the present status of cGAS–STING agonists, emphasizing preclinical and clinical advances in combination therapy with ICIs, and discusses the challenges and future directions to optimize efficacy, improve safety, and expand the therapeutic potential of this strategy in oncology.

## Introduction

Immune checkpoint inhibitors (ICIs) targeting programmed death-1 (PD-1), programmed death ligand-1 (PD-L1), and cytotoxic T lymphocyte antigen-4 (CTLA-4) have transformed the therapeutic landscape for multiple malignancies, including melanoma, non-small cell lung cancer (NSCLC), triple-negative breast cancer (TNBC), renal cell carcinoma, and Hodgkin lymphoma [1–4]. Physiologically, the PD-1/PD-L1 axis functions as a key immune checkpoint, with PD-L1 on antigen-presenting cells (APCs) and tissue cells binding PD-1 on activated T cells to restrain excessive activation, maintain peripheral tolerance, and prevent autoimmunity [5]. Tumors frequently exploit this pathway by aberrantly upregulating PD-L1 to engage PD-1, thereby suppressing CTL function and evading immune surveillance [6]. While the PD-1/PD-L1 axis is one of the best-characterized checkpoints, CTLA-4 and others also contribute to immune homeostasis, and their dysregulation within the tumor microenvironment (TME) further amplifies immunosuppression. By blocking inhibitory immune pathways, ICIs restore cytotoxic T lymphocyte (CTL) activity, enabling durable antitumor responses in subsets of patients. Despite these successes, objective response rate (ORR) remain modest—often below 40%—and both primary and acquired resistance are common [7–9].

One critical factor restricting ICI efficacy is the immunological profile of the TME. According to the degree of immune cell infiltration and inflammatory activity, tumors are divided into T cell–inflamed (“hot”) and non–T cell–inflamed (“cold”) [10]. “Cold” tumors are defined by limited effector T cell presence, impaired antigen presentation, defective type I interferons (IFN-I) signaling, and an enrichment of inhibitory immune subsets including regulatory T cells (Tregs), myeloid-derived suppressor cells (MDSCs), and M2-skewed tumor-associated macrophages (TAMs) [11, 12]. In such contexts, ICIs lack sufficient effector immune cells to unleash, resulting in suboptimal therapeutic outcomes.

**Fig. 1:**
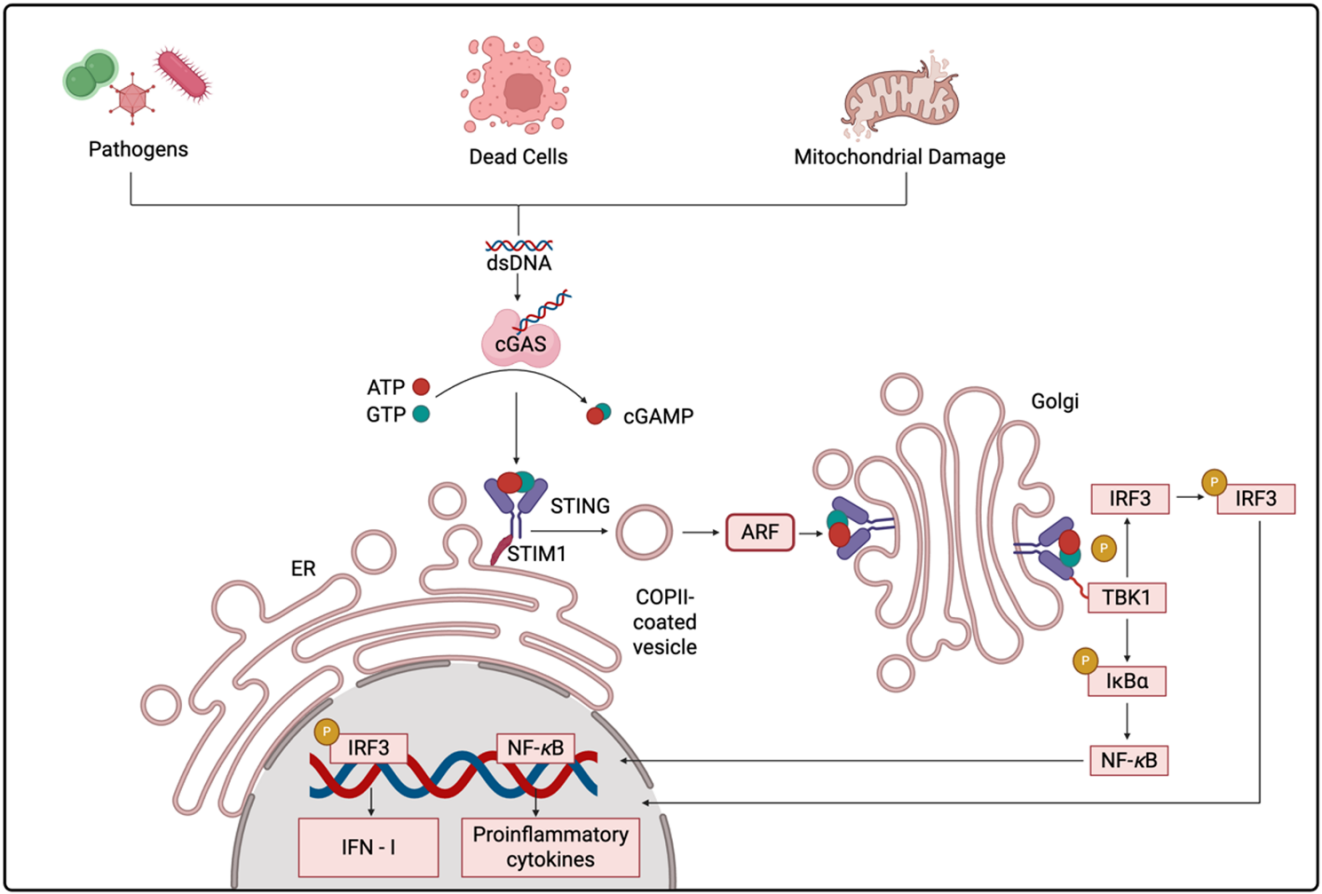
Diagram of the cGAS–STING signaling cascade triggered by cytosolic DNA. Pathogen invasion, release of DNA from dead cells, or mitochondrial damage can result in cytoplasmic accumulation of dsDNA. Cytosolic dsDNA is recognized by cyclic cGAS, mediating the transformation of ATP and GTP into cGAMP. The generated cGAMP interacts with the adaptor protein STING, located on the ER membrane, and releases STING from its association with STIM1. Activated STING undergoes conformational changes, oligomerization, and is transported to the Golgi through COPII-coated vesicles in an ARF-dependent fashion. Within the Golgi, STING engages TBK1, which subsequently phosphorylates IRF3 and IκBα. Once phosphorylated, IRF3 forms dimers and relocates into the nucleus to induce IFN-I expression, while IκBα degradation releases NF-κB to promote transcription of proinflammatory cytokines. This signaling cascade links innate and adaptive immune responses, thereby contributing to anti-tumor immunity

In 2013, Chen et al. made a landmark discovery by identifying cyclic GMP–AMP synthase (cGAS) in mammalian cells and demonstrating that it catalyzes the formation of cGAMP, a pivotal second messenger capable of directly engaging and activating stimulator of interferon genes (STING) [13]. This breakthrough established the cGAS–STING axis as a key modulator of innate immune responses, which has subsequently driven intensive studies on its biological and disease-related functions [14, 15]. Mechanistically, when double-stranded DNA (dsDNA) originating from pathogens, injured host cells, or tumor-derived micronuclei is detected, cGAS facilitates the production of the second messenger 2’3’-cyclic GMP–AMP (2′3′-cGAMP) using ATP and GTP [16, 17]. The newly produced 2’3’-cGAMP interacts with STING, an adaptor anchored in the endoplasmic reticulum (ER) membrane, initiating profound conformational changes. Activated STING oligomerizes and traffics from the ER to the Golgi apparatus through COPII vesicles, where it undergoes palmitoylation at cysteine residues essential for full signaling activity [18, 19]. TBK1 phosphorylates STING and interferon regulatory factor 3 (IRF3), leading to IRF3 dimerization, nuclear translocation, and transcription of IFN-I. Concurrently, activation of NF-κB drives proinflammatory cytokine and chemokine production, including CXCL9 and CXCL10 [20] (Fig. 1). Through induction of IFN-I, dendritic cell (DC) maturation, and enhanced antigen cross-presentation, cGAS–STING signaling serves as a crucial bridge between innate and adaptive immunity [21, 22]. It enhances the infiltration and functional activation of effector CD8^+^T cells, boosts the activity of natural killer (NK) cells, and reprograms TAMs toward a proinflammatory M1 phenotype [23]. These actions may transform “cold” tumors into “hot” ones with robust immune infiltration, a phenotype more responsive to ICI therapy [24].

Preclinical studies have shown that pharmacological STING agonists—including synthetic cyclic dinucleotide (CDN) analogs, non-nucleotide small molecules, and nanoparticle-based delivery systems—synergize with synergize with PD-1/PD-L1 inhibition to induce strong and durable anti-tumor immunity in models of TNBC (4T1) [25]. Preclinical studies of STING agonists exemplified by MK-1454 have demonstrated strong cytokine induction and anti-tumor effects in syngeneic murine models, where pairing with PD-1 inhibition further enhanced tumor regression. These findings laid the foundation for the clinical advancement of STING agonists used alongside PD-1/PD-L1 inhibitors [26].

In this review, we provide a comprehensive overview of the mechanistic basis underlying cGAS–STING pathway activation in remodeling the TME and enhancing ICI responses. We highlight preclinical and clinical evidence supporting the use of STING agonists in combination with ICIs, discuss pharmacological and translational challenges, and propose future research directions. Our objective is to establish a mechanistic and translational framework for integrating cGAS–STING activation into cancer immunotherapy strategies to overcome resistance to ICIs.

## Outline of the cGAS–STING signaling pathway

Extensive research over the past decade has established the cGAS–STING axis as a central regulator of innate immunity and tumor immune responses [27]. In the context of cancer immunotherapy, cGAS–STING–mediated signaling promotes IFN-I production and chemokine release, thereby fostering an immunologically active TME [28, 29]. This section summarizes the mechanistic basis of the pathway, highlights its diverse roles in shaping the TME, and discusses its promise as a target for next-generation cancer immunotherapy.

### Pathogen invasion and cytosolic DNA mislocalization as drivers of cGAS activation

cGAS is a DNA-sensing enzyme that belongs to the nucleotidyltransferase (NTase) superfamily, characterized by an unstructured N-terminal region together with a defined C-terminal catalytic domain [30]. It serves as a molecular sentinel for aberrant dsDNA appearing in the cytoplasm, regardless of sequence origin [16, 31]. Such sensing usually arises under two pathological conditions: infection by DNA-bearing pathogens—such as specific viruses, bacteria, or retroviruses that deliver exogenous DNA into the cytosol—or cellular injury causing nuclear or mitochondrial DNA to leak into the cytoplasm [32, 33].

High-resolution crystallographic and biochemical investigations have revealed the architectural principles of this activation process. The C-terminal NTase domain, which performs the catalytic function, is organized into three essential modules: a DNA-binding interface, the enzymatic catalytic center, and zinc-binding structural motifs [34]. Zinc ions play a critical scaffolding role, tethering dsDNA to a pair of cGAS molecules and driving the assembly of a 2:2 cGAS–dsDNA complex, which switches the enzyme into an active conformation [35]. Within this complex, the cGAS dimers orient head-to-head along the DNA, giving rise to a characteristic ladder-like framework that can trace the contour of a curved DNA helix or bridge separate DNA strands [36, 37]. This arrangement markedly strengthens the overall stability of the DNA–protein complex [38].

Importantly, cGAS activation is not dictated solely by its catalytic region. The flexible N-terminal domain, enriched in basic amino acids, exerts additional control through liquid–liquid phase separation (LLPS). In concert with zinc ions and extended dsDNA segments, it condenses into dynamic, membrane-less assemblies [39]. This condensation is highly sensitive to DNA concentration, occurring only when the amount of cytosolic dsDNA rises above a threshold level. Such a mechanism imposes stringent spatial and temporal regulation, ensuring that the cGAS pathway is triggered exclusively under conditions of significant DNA mislocalization [40].

### The cGAS–STING axis: orchestrating immune activation in the TME

The ability of DNA to provoke immune reactions was recognized well before it was confirmed as the genetic material [41]. In healthy eukaryotic cells, DNA is compartmentalized within the nucleus and mitochondria, thereby avoiding direct contact with the cytosol and preventing inappropriate immune activation [42]. In malignant cells, however, multiple stressors can disrupt this segregation. Endogenous drivers such as chromosomal instability, oxidative damage, along with exogenous insults including chemotherapy and radiotherapy, can cause fragments of nuclear DNA or mitochondrial DNA (mtDNA) to leak into the cytoplasm. This mislocalization acts as a danger signal that activates the cGAS–STING signaling axis [43–45].

Once triggered, STING undergoes oligomerization, which subsequently triggers multiple downstream signaling cascades—chiefly the induction of IRF3- and NF-κB-regulated transcriptional pathways [46]. Upon activation, IRF3 dimerizes and translocates into the nucleus, driving the transcription of IFN-I, TNF, and IL-6. Among these, IFN-I plays a central role in coordinating anti-cancer immunity. It facilitates T-cell priming against tumor-associated antigens (TAA), augments DC recruitment and retention in the tumor milieu, and strengthens cross-presentation of tumor-derived antigens to cytotoxic T lymphocytes [47]. By coordinating these processes, the cGAS–STING pathway functions as an essential link connecting innate and adaptive immunity, thereby enabling the establishment of strong and lasting anti-tumor responses [48].

### Synergistic mechanisms of cGAS–STING activation with immune checkpoint inhibitors

Combining cGAS–STING pathway stimulation with immune checkpoint inhibition produces synergistic benefits, as both contribute complementary mechanisms to the initiation and persistence of antitumor immune responses. Although ICIs lift inhibitory signals on T cells, their therapeutic effect depends on tumor-reactive T cells being located in the TME. Many tumors fail to respond to ICIs because they lack a pre-existing T cell infiltrate—a hallmark of immunologically “cold” tumors. By activating innate immune pathways and promoting type I IFN responses, cGAS–STING signaling can convert these tumors into “hot” tumors that are more responsive to checkpoint blockade.

### Turning “Cold” tumors into “Hot” tumors

Effective antitumor immunity critically depends on the presence and functionality of tumor-infiltrating lymphocytes (TILs), particularly cytotoxic CD8⁺ T cells and helper CD4⁺ T cells. In immunologically “cold” tumors, characterized by scarce T cell infiltration and weak inflammatory signaling, the cGAS–STING pathway is crucial for transforming these tumors into “hot” phenotypes enriched with immune effector cells.

Accumulating evidence indicates that STING activation is closely tied to the effector roles of CD4^+^ and CD8^+^T cells [49, 50]. Within CD4^+^T cells, STING signaling induces differentiation toward Th1 and Th9 subsets and enhances secretion of cytokines such as interferon-gamma (IFN-γ) and interleukin-9 (IL-9) [49]. Mechanistically, cGAMP-mediated STING activation triggers IRF3 phosphorylation and IFN-I secretion, driving Th1 polarization. In parallel, cGAMP can trigger the mTOR pathway, leading to phosphorylation of downstream molecules such as p70S6 kinase and ribosomal protein S6, which promotes Th9 differentiation and enhances IL-9 secretion [49]. In CD8^+^ T cells, STING signaling is critical for sustaining the TCF1^+^ phenotype, supporting long-term survival and secondary immune responses [50]. Expression of TCF1 is further enhanced via cGAS–STING–dependent IFN-I signaling, ensuring robust proliferation and activity of tumor-specific CD8^+^T cells.

Efficient T cell–mediated tumor eradication also requires their recruitment from circulation into the TME. STING signaling in DCs drives strong expression of the chemokines CXCL9 and CXCL10 [51–53]. hese chemokines function to recruit effector CD8^+^T cells but also recruit NK cells, which directly kill tumor cells and enhance dendritic cell infiltration into tumors. Similarly, in tumor endothelial cells, STING activation triggers type I IFN production, which in turn stimulates CXCL10 secretion, facilitating T cell transendothelial migration [54, 55]. Moreover, STING signaling enhances the expression of adhesion molecules such as E-selectin, vascular cell adhesion molecule-1 (VCAM-1), and intercellular adhesion molecule-1 (ICAM-1) on tumor endothelial cells, thereby facilitating T cell extravasation [56, 57]. Beyond chemokine induction, STING activation contributes to tumor vessel normalization by increasing pericyte coverage and enhancing adhesion molecule expression [57, 58]. This vascular remodeling alleviates hypoxia, improves immune cell infiltration, and sustains an inflamed TME conducive to effective immune checkpoint blockade therapy.

**Fig. 2:**
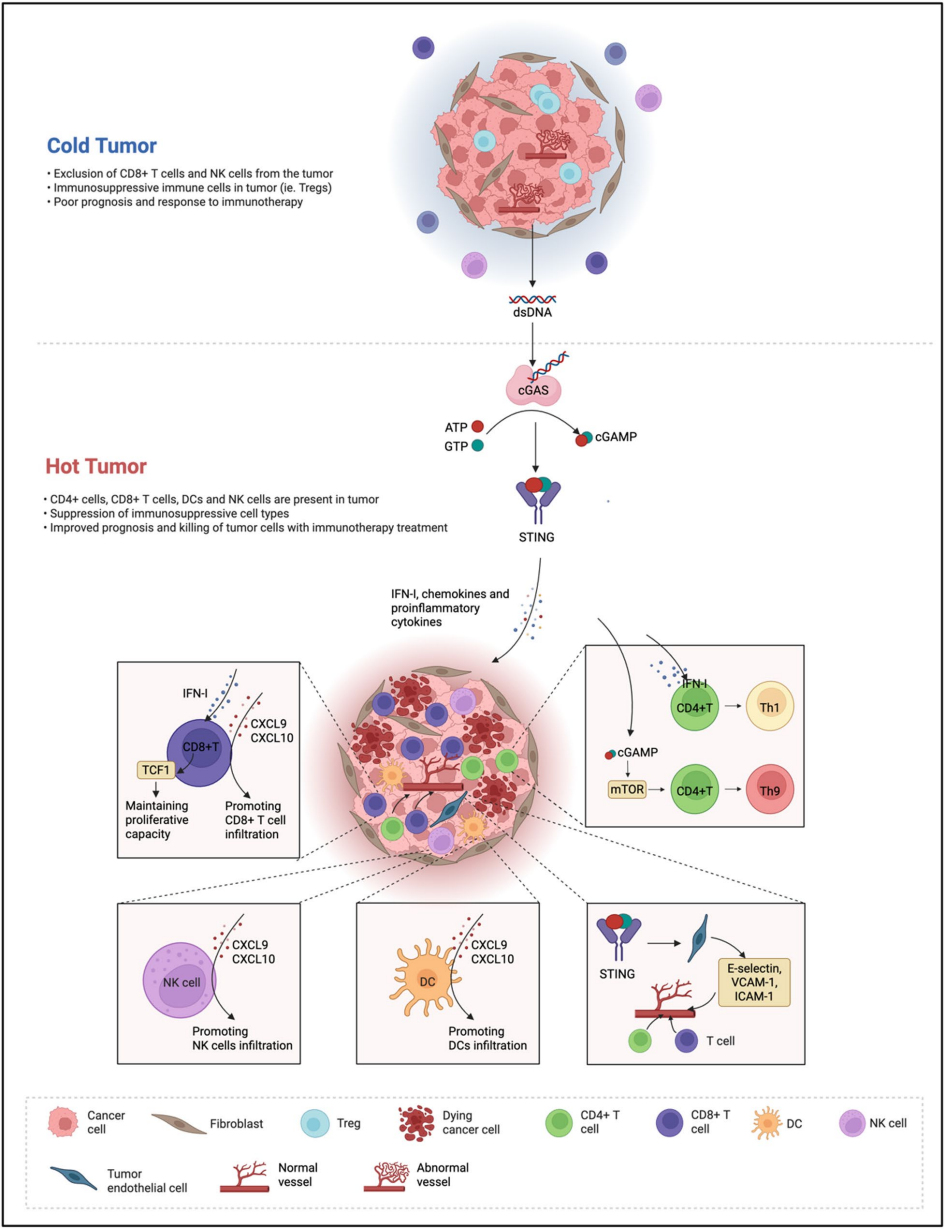
Schematic representation of cGAS–STING pathway–mediated conversion of “cold” tumors into “hot” tumors. cGAS–STING signaling transforms a “cold” tumor into a “hot” tumor by inducing IFN-I, chemokines, and proinflammatory cytokines. This promotes CD8⁺ T cell proliferation and infiltration, Th1/Th9 differentiation of CD4⁺ T cells, NK cell and DC recruitment, and vascular normalization through upregulation of adhesion molecules, thereby enhancing immune cell infiltration and responsiveness to immunotherapy

Collectively, these findings indicate that cGAS–STING pathway activation can reprogram the immunosuppressive TME by boosting T cell functionality, enhancing chemokine-driven immune cell recruitment, and promoting vascular normalization—key processes in transforming “cold” tumors into “hot” tumors responsive to immunotherapy (Fig. 2).

#### Enhancing antigen presentation and T cell priming

The success of ICIs is contingent upon a sufficient reservoir of tumor-specific T cells that are effectively activated. Robust antigen presentation together with efficient priming of T cells underpins the initiation and persistence of durable antitumor responses. Inadequate priming or insufficient antigen presentation limits the ability of ICIs to elicit meaningful CTL activity [59–61]. Stimulation of the cGAS–STING signaling cascade in APCs promotes interferon release and upregulation of costimulatory factors, which in turn significantly strengthens antigen presentation and T cell priming, acting synergistically with ICI therapy [62, 63].

DCs, recognized as the most efficient APCs, exhibit superior ability to capture, process, and present tumor antigens. After antigen capture, DCs traffic to draining lymph nodes, where tumor peptides are displayed on MHC-I molecules for naïve CD8^+^T cells, triggering adaptive immunity [64]. DCs engulf tumor-derived DNA, which is detected in the cytosol by cGAS. This recognition activates STING signaling, triggering strong IFN-I production and promoting DC maturation [65]. In addition, tumor-secreted cGAMP can be directly transferred to DCs, bind STING, and amplify IFN-driven maturation signals [66]. Exosomes serve as another important vehicle for communication between tumor cells and immune cells [67]. Tumor-derived exosomes can carry dsDNA into DCs, triggering cGAS–STING activation, which promotes IFN-I secretion and elevates CD40, CD80, and CD86 expression, ultimately promoting T cell responses. Irradiated tumor cell–derived exosomes display strong immunogenic activity, inducing IFN-related genes (IFN-β, Mx1, IFNAR1) in DCs and stimulating the release of IFN-β, IL-6, and CXCL10 [68], which together promote an inflammatory niche that supports T cell priming. Recently, receptor-mediated endocytosis has gained attention as a valuable method for improving antigen transport into DCs. For example, targeting DC-specific membrane receptors such as CD11c with monoclonal antibodies can significantly enhance antigen uptake and processing. When combined with STING activation, this strategy further promotes DC maturation and their capacity to prime T cells [69, 70].

**Fig. 3:**
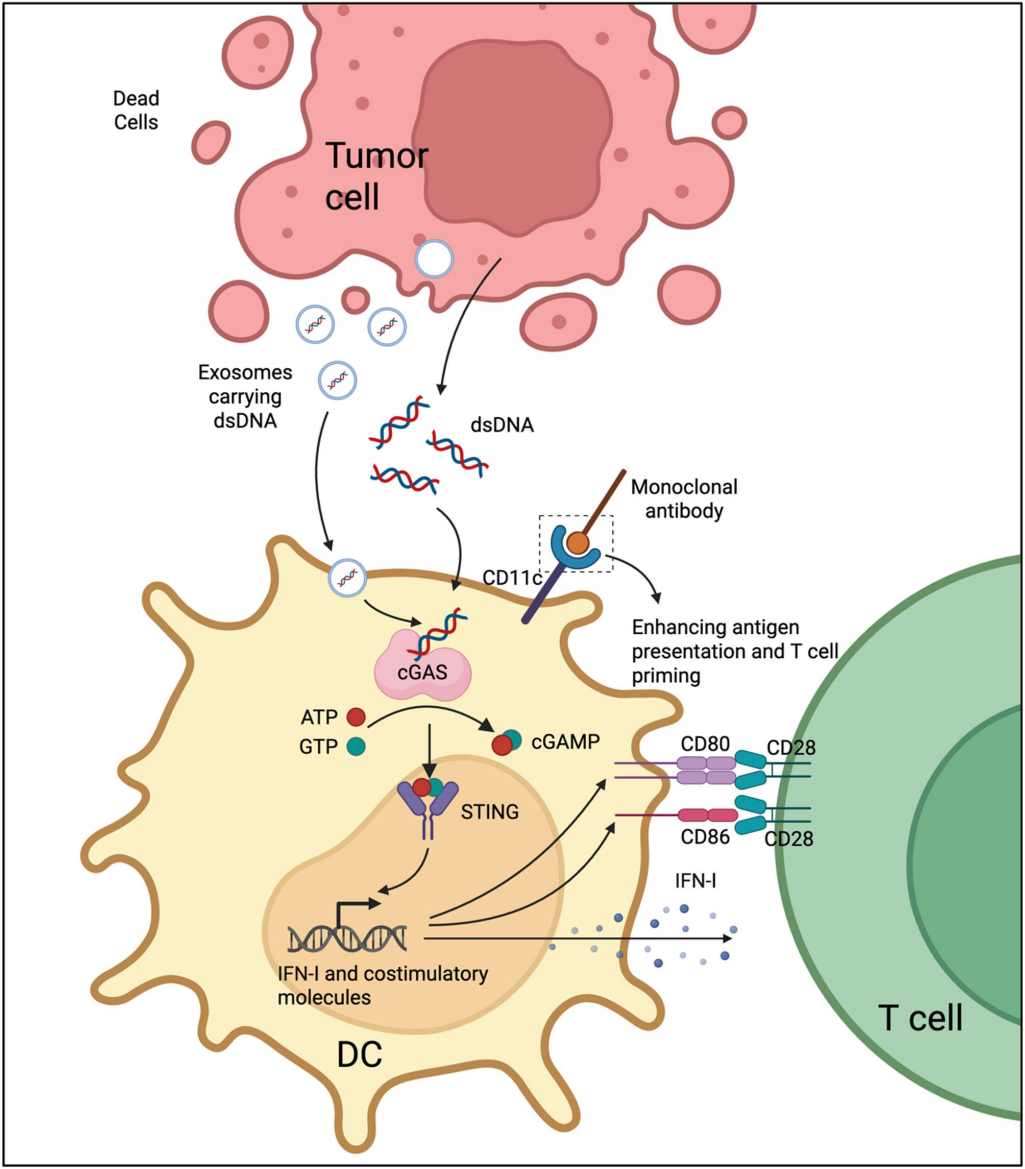
Schematic illustration of the cGAS–STING signaling pathway in DCs enhancing antigen presentation and T cell priming. DsDNA released from dying tumor cells or delivered via tumor-derived exosomes is taken up by DCs and recognized in the cytosol by cGAS, which catalyzes the synthesis of cGAMP from ATP and GTP. cGAMP activates STING, leading to robust induction of IFN-I and upregulation of costimulatory molecules such as CD80 and CD86, thereby enhancing antigen presentation and T cell activation through CD28-mediated costimulation. In addition, receptor-targeted strategies, such as monoclonal antibodies against CD11c, further improve antigen uptake, DC maturation, and T cell priming

Collectively, activation of the cGAS–STING pathway in dendritic cells enhances antigen presentation capacity, upregulates costimulatory molecule expression, and drives potent IFN-I secretion, thereby facilitating effective T cell priming. This process provides a stronger immunological foundation for ICIs, maximizing both the magnitude and durability of antitumor responses (Fig. 3).

#### Remodeling the immunosuppressive TME

The clinical activity of ICIs is often hindered by the presence of an immunosuppressive TME enriched in TAMs, MDSCs, and Tregs [71]. These cell subsets suppress CTL responses through direct inhibitory signaling, secretion of immunosuppressive cytokines, and modulation of tumor vasculature, ultimately enabling tumor immune evasion [72]. Even in tumors with pre-existing T cell infiltration, persistent immunosuppressive networks can attenuate responses to PD-1/PD-L1 or CTLA-4 blockade [73, 74]. Consequently, effective reprogramming or depletion of these inhibitory immune populations represents a critical approach to improve ICI efficacy.

The cGAS–STING pathway has emerged as a key regulator capable of reshaping the immunosuppressive TME. Its second messenger, 2′3′-cGAMP, can be transferred between cells via solute carrier (SLC) family transporters, including SLC19A1 and SLC46A2, allowing uptake by monocytes and macrophages [75–77]. This intercellular trafficking of cGAMP fine-tunes STING activation in immune and stromal compartments, influencing immune cell composition and function within the TME. Experimental depletion of extracellular cGAMP significantly reduced the proportion of CD11c⁺ DCs and CD103⁺CD11c⁺ conventional type 1 DCs (cDC1s) within the CD45⁺ MHC II⁺ APC compartment, underscoring its role in sustaining effective antigen presentation [78].

In peritoneal colon cancer, tumor-derived cues drive the accumulation of M2-like macrophages, which suppress effector T cell activity during metastatic dissemination [57]. STING agonists counteract this process by reprogramming TAMs toward an M1-like phenotype, characterized by upregulation of proinflammatory genes and downregulation of M2-associated transcripts, while simultaneously normalizing tumor vasculature through enhanced pericyte coverage and increased adhesion molecule expression, thereby promoting CD8⁺ T cell infiltration [57].

Beyond TAM reprogramming, STING activation suppresses MDSC expansion and inhibitory functions. Mechanistically, it induces suppressor of cytokine signaling 1 (SOCS1) expression in tumor cells and MDSCs, reducing immunosuppressive cytokine production and promoting chemokine secretion that facilitates monocyte, macrophage, and T cell recruitment to the tumor site [79–81]. STING activation also diminishes Treg abundance in the TME, further enhancing antitumor immunity [82].

In addition, cGAS–STING signaling modulates other immune components relevant to ICI responsiveness. Vascular endothelial cell–intrinsic STING activation upregulates CCR5 on CD8⁺ T cells and induces CCL5 production, promoting the development of tertiary lymphoid structures (TLSs) [83]. STING-activated CD11c⁺ DCs further augment TLS formation through increased expression of lymphotoxin-α (LTA), interleukin-36 (IL-36), inflammatory chemokines, and type I interferons. LTA serves as a key driver of lymphoid neogenesis, whereas IL-36 facilitates the recruitment and activation of T and B lymphocytes within the tumor milieu, and together these mediators promote the development of TLSs [84]. Nevertheless, STING activation may also drive the expansion of regulatory B cells (Bregs) in certain contexts, which can impair NK cell-mediated tumor clearance [85], indicating that combinatorial strategies should carefully consider these potential counteractive effects.

Collectively, these findings illustrate that cGAS–STING activation can reprogram the immunosuppressive TME by modulating myeloid cell phenotypes, normalizing vasculature, depleting inhibitory cell subsets, and fostering TLS formation. Such TME remodeling supports the infiltration and function of effector T cells, thereby potentiating the clinical benefit of immune checkpoint blockade.

#### Preclinical and clinical advances in cGAS–STING and ICI combination therapy

The concept of combining cGAS–STING activation with immune checkpoint inhibition has gained momentum over the last decade, supported by robust preclinical evidence and an expanding portfolio of clinical trials. The primary goal is to exploit the immunostimulatory effects of STING activation to improve tumor immunogenicity, thereby enhancing the efficacy of ICIs across a broad spectrum of cancers.

#### Preclinical studies

PD-1 is a pivotal inhibitory receptor expressed on activated T cells that plays a central role in maintaining immune tolerance. Its ligand, PD-L1, is often upregulated by tumor cells, where it binds PD-1 and transmits inhibitory signals that suppress T cell activation. This PD-1/PD-L1 interaction dampens CTL activity, enabling tumor cells to evade immune surveillance. Monoclonal antibodies targeting PD-1 or PD-L1 disrupt this suppressive signaling axis, thereby restoring T cell functionality and antitumor activity. Agonists of the STING pathway can potentiate this effect by promoting T cell activation, proliferation, and infiltration into tumors. By triggering IFN- I production and enhancing antigen presentation, STING agonists act as effective immune sensitizers that complement PD-1/PD-L1 blockade. The presence of pre-existing mature CTLs within the tumor microenvironment is a key determinant of successful anti-PD-1/PD-L1 therapy. Notably, ICIs can counterbalance certain immunoregulatory effects induced by STING pathway activation [86, 87]. An example is the SatVax vaccine, which incorporates cGAMP with antigenic peptides (Q19D and Q15L). In combination with anti-PD-L1 therapy, SatVax markedly increased the frequency of E7-specific CD8⁺ T cells, while reducing the proportion of exhausted phenotypes (CD8⁺Tim3⁺ and CD8⁺PD-1⁺), leading to complete tumor clearance in four out of five treated mice [86]. Similarly, poly (β-amino ester) (PBAE) nanoparticles enhance the delivery of CDNs, and when co-administered with anti-PD-1 antibodies, they substantially delayed tumor progression in B16 melanoma models compared with either treatment alone [87]. Furthermore, in various murine xenograft models, combined STING agonist and anti-PD-1 therapy conferred protection against tumor rechallenge [88, 89]. In a B16-F10 melanoma lung metastasis model, anti-PD-1 monotherapy showed minimal efficacy; however, co-treatment with STING-lipid nanoparticles (STING-LNP) produced synergistic antitumor effects. STING-LNP administration significantly upregulated CD3, CD4, NK1.1, PD-1, and IFN-γ expression within metastatic lesions. Mechanistically, this was driven by IFN-I production from liver macrophages that had internalized STING-LNP, leading to systemic activation of PD-1⁺ NK cells. The resulting IFN-γ induced PD-L1 expression on tumor cells, thus leading to a synergistic antitumor effect when anti-PD-1 is administered [90]. These studies collectively demonstrate that while STING agonists can markedly enhance antigen presentation, promote dendritic cell maturation, and activate CD8⁺ T cells, their efficacy as monotherapies remains limited. On one hand, STING-mediated type I interferon signaling facilitates cross-presentation and effector T cell recruitment, laying the foundation for antitumor immunity. On the other hand, STING activation concurrently induces immunoregulatory cytokines, including IFN-γ and IL-6, which upregulate PD-L1 expression on both tumor and immune cells. Elevated PD-L1 then suppresses tumor-infiltrating CTL activity, establishing a negative feedback loop that constrains the durability of STING-driven immune responses. Therefore, combining STING agonists with PD-1/PD-L1 blockade not only leverages their capacity to boost antigen presentation and T cell priming but also circumvents the counter-regulatory induction of PD-L1, ultimately achieving stronger and more durable antitumor effects.

In addition to PD-1/PD-L1 inhibition, STING activation also enhances the efficacy of CTLA-4 blockade. Anti-CTLA-4 therapy functions by lowering the activation threshold of T cells, promoting robust tumor-specific immune responses [91]. Harding et al. demonstrated that an intact cGAS–STING pathway is required for optimal anti-CTLA-4 activity. In B16 melanoma models subjected to radiotherapy and tumor antigen exposure, subsequent anti-CTLA-4 treatment markedly improved tumor clearance, whereas STING-deficient tumors exhibited reduced responsiveness and diminished CD8⁺ T cell infiltration [92]. Further, Ager et al. assessed the effects of triple checkpoint blockade (anti-CTLA-4, anti-PD-1, and anti-4-1BB) in prostate cancer models. While this combination induced regression of bilateral tumors in 40% of animals, the addition of the STING agonist cyclic diguanylate monophosphate (CDG) increased complete regression rates to 75% [93]. Local co-delivery of CDG with ICIs expanded the repertoire of tumor-infiltrating CD8⁺ T cells and elevated the proportion of stimulator of prostatic adenocarcinoma specific (SPAS)-specific T cells, thereby broadening antitumor immunity and enhancing responses to subdominant tumor antigens.

#### Clinical trials of STING agonist in combination with ICI therapy

Given the ability of STING activation to enhance antigen presentation, promote T cell infiltration, and reprogram the immunosuppressive tumor microenvironment, combining STING agonists with ICIs has emerged as a promising therapeutic strategy. Early-phase clinical trials have explored a variety of STING agonist modalities—ranging from CDNs to non-CDN small molecules—in combination with PD-1/PD-L1 or CTLA-4 blockade. CDN agonists, such as ADU-S100 and ulevostinag, mimic endogenous second messengers like cGAMP but generally require intratumoral injection due to limited systemic stability. In contrast, non-CDN agonists, including dazostinag and MK-2118, are chemically distinct small molecules with more favorable pharmacokinetic properties, allowing systemic administration and broader clinical applicability [94]. These structural and pharmacological differences are critical for interpreting trial outcomes and are discussed in the following subsections (Table 1).

#### CDN agonists

CDN STING agonists are a novel class of immunotherapeutic agents capable of enhancing the efficacy of ICIs by inducing IFN- I production, upregulating costimulatory molecule expression, and promoting effective T cell priming. Several CDN-based STING agonists have entered clinical evaluation, among which ulevostinag and MIW815 (ADU-S100) are the most extensively studied.

ADU-S100 (also known as MIW815) is a synthetic CDN designed to resist degradation by phosphodiesterases and exhibits higher binding affinity to both human and murine STING compared with unmodified CDNs [22]. In preclinical studies, intratumoral administration of ADU-S100 elicited robust CD8⁺T cell responses and promoted regression of esophageal adenocarcinoma [95]. These findings provided the rationale for advancing ADU-S100 into clinical testing. Owing to its promising antitumor activity, ADU-S100 has advanced into clinical testing. In a phase Ib multicenter dose-escalation study (NCT03172936), 106 patients with advanced solid tumors or lymphomas received intratumoral MIW815 in combination with a fixed dose of spartalizumab. The combination was well tolerated, with the most common adverse events (AEs) being pyrexia (22%), injection site pain (20%), and diarrhea (11%), and the maximum tolerated dose was not reached. Pharmacodynamic analyses confirmed effective on-target STING activation. However, the ORR was only 10.4%, and no meaningful clinical benefit was observed in patients with PD-1–refractory disease [96].

Another CDN-based STING agonist, MK-1454, developed by Merck, exhibits high affinity for STING in vitro and strongly induces IFN-β secretion [26]. These preclinical findings, including tumor regression and synergy with anti–PD-1 therapy in murine models [26], provided the rationale for clinical translation. Its clinical candidate, ulevostinag, was evaluated in a phase I dose-escalation and expansion trial (NCT03010176) involving patients with advanced or metastatic solid tumors or lymphomas who received intratumoral ulevostinag as monotherapy or in combination with intravenous pembrolizumab. The treatment was well tolerated, with pyrexia being the most common AE. Pharmacodynamic profiling revealed marked increases in pro-inflammatory cytokines, including CXCL10, IFN-γ, and IL-6, indicating effective STING pathway activation [97]. In a subsequent randomized phase 2 trial (NCT04220866) in patients with untreated metastatic or unresectable, recurrent head and neck squamous cell carcinoma (HNSCC), the combination of ulevostinag with pembrolizumab achieved higher ORR than pembrolizumab monotherapy, suggesting synergistic antitumor activity [98].

Overall, CDN STING agonists combined with PD-1 blockade are well tolerated across different tumor types and can induce clear immunologic activation; however, their clinical antitumor activity appears variable. Ulevostinag has shown higher response rates in specific settings, such as ICI-naïve HNSCC, whereas MIW815 has demonstrated more limited efficacy in a broader range of solid tumors. Future research should focus on precise patient selection, optimization of delivery routes, and rational combination strategies with other immunomodulatory agents to maximize clinical benefit.

#### Non-CDN STING agonists

Non-CDN STING agonists have also been explored in combination with ICIs to overcome the poor responsiveness of certain solid tumors to PD-1/PD-L1 blockade. Dazostinag, a novel non-CDN STING agonist, has demonstrated potential immune-activating effects in adenoid cystic carcinoma (ACC), a rare and highly lethal malignancy with extremely low response rates to cytotoxic chemotherapy and ICIs. Comprehensive immune profiling revealed that ACC tumors are immunologically “cold,” characterized by sparse T cell infiltration, low PD-L1 expression, and markedly reduced beta-2-microglobulin (B2M) and HLA class I expression—features likely contributing to immune evasion. In vitro treatment of freshly resected primary ACC tissues with interferon-γ or a STING agonist strongly upregulated HLA-I/B2M expression. Clinically, in a patient with recurrent, metastatic breast ACC, dazostinag combined with pembrolizumab achieved a partial response with approximately 70% tumor reduction, suggesting that STING pathway activation can reverse antigen presentation defects and restore ICI sensitivity in such tumors (NCT04420884) [99].

Another non-CDN STING agonist, MK-2118, has undergone clinical evaluation in the first-in-human phase I trial (NCT03249792) in patients with refractory, advanced solid tumors or lymphomas. Participants received intratumoral (IT) MK-2118 monotherapy, IT MK-2118 plus intravenous pembrolizumab, or subcutaneous (SC) MK-2118 plus pembrolizumab. The IT dosing ranged from 100 to 20,000 µg, and SC dosing from 5,000 to 150,000 µg, with no maximum tolerated dose identified. Grade 3/4 treatment-related AEs occurred in 22%, 23%, and 11% of participants in the respective arms, indicating manageable safety. Pharmacokinetic analysis revealed dose-dependent systemic exposure for both IT and SC routes; however, only IT administration induced systemic immune responses, including dose-dependent increases in STING-related blood RNA expression, IFN-γ, IFN-γ–induced protein 10, and IL-6. SC administration did not produce dose-related immune effects. ORR were 0%, 6%, and 4% in the three arms, respectively [100].

Collectively, these findings suggest that non-CDN STING agonists combined with PD-1 blockade are generally well tolerated and can induce immune activation in certain contexts, particularly in immunologically cold tumors through restoration of antigen presentation and T cell responses. However, their clinical antitumor efficacy remains modest, underscoring the need for further research to optimize delivery routes, dosing regimens, and patient selection strategies.

**Table 1:** Clinical trials of STING agonist in combination with ICI therapy

cGAS-STING drug	Combination drug	STING agonist type	Cancer type	Phase	NCT number	ORR	PFS/OS	AEs
MIW815(ADU-S100)	Pembrolizumab	CDN	Head and Neck Cancer	Phase II	NCT03937141	Ongoing		
MIW815(ADU-S100)	Spartalizumab	CDN	Advanced solid tumors or lymphomas	Phase I	NCT03172936	10.4%	Not reported	Pyrexia (22%), injection site pain (20%), diarrhea (11%)
Ulevostinag (MK-1454)	Pembrolizumab	CDN	Advanced or metastatic solid tumors or lymphomas	Phase I	NCT03010176	12%	Not reported	Pyrexia (70%)
MK-1454	Pembrolizumab	CDN	HNSCC	Phase II	NCT04220866	50%	Median PFS (6.4 months)	Pyrexia (62.5%)
GSK3745417	Dostarlimab	Non-CDN	Advanced solid tumors	Phase I	NCT03843359	Ongoing		
SNX281	Pembrolizumab	Non-CDN	Advanced solid tumors or lymphomas	Phase I	NCT04609579	Ongoing		
Dazostinag (TAK-676)	Pembrolizumab	Non-CDN	Advanced or Metastatic Solid Tumors	Phase I/II	NCT04420884	1/1 PR (≈ 70% tumor reduction)	Not reported	Not reported
Dazostinag (TAK-676)	Pembrolizumab	Non-CDN	NSCLC, TNBC and HNSCC	Phase I	NCT04879849	Ongoing		
MK-2118 (IT/SC)	Pembrolizumab	Non-CDN	Advanced/Metastatic Solid Tumors or Lymphomas	Phase I	NCT03249792	6% (IT MK-2118), 4% (SC MK-21)	IT: Median PFS (2.0 months)/Median OS (9.7 months); SC: PFS (1.9 months)/Median OS (9.4 months)	IT: 23%; SC: 11%
TAK-500	Pembrolizumab	Antibody–drug conjugate	Select Locally Advanced or Metastatic Solid Tumors	Phase I/II	NCT05070247	Ongoing		

#### Challenges and limitations of cGAS–STING + ICI combination therapy

Despite compelling preclinical evidence and encouraging early-phase clinical trial results, the integration of cGAS–STING agonists with ICIs faces substantial biological and translational hurdles. The complexity of cGAS–STING signaling, coupled with tumor-intrinsic resistance mechanisms, an immunosuppressive TME, and the risk of exacerbated immune-related toxicities, presents formidable barriers to clinical success. Understanding these multifaceted challenges is essential for designing rational combination strategies that maximize therapeutic efficacy while minimizing adverse effects. The following subsections outline key resistance pathways, TME-mediated suppression, and safety concerns that must be addressed to realize the full potential of this approach.

#### Tumor-Intrinsic resistance mechanisms

The cGAS–STING pathway can exert both antitumor and protumor effects depending on the tumor’s genetic background and microenvironmental context [101]. This context dependency represents a major source of intrinsic resistance to STING agonists and ICIs. Under conditions such as hypoxia, nutrient deprivation, or specific immune cell compositions, pathway activation may fail to sustain cytotoxic immune responses and instead promote immunosuppressive programs, thereby reducing therapeutic efficacy [102]. For example, in HPV⁺ tongue squamous cell carcinoma, STING activation was found to strongly induce CCL22, IL-10, and indoleamine 2,3-dioxygenase (IDO) expression, which together promoted the recruitment and expansion of Foxp3⁺ Tregs within the tumor stroma. Patient tissue analyses confirmed that high STING activity correlated with elevated CCL22 levels and increased Treg infiltration, while mechanistic studies revealed that CCL22 upregulation was driven by c-Jun signaling and negatively regulated by miR-27. This STING–CCL22–Treg axis created an immunosuppressive microenvironment that curtailed antigen-specific CD8⁺ T-cell proliferation and effector function, ultimately limiting antitumor responses to STING agonists [103].

A distinct form of intrinsic resistance is observed in tumors with high chromosomal instability (CIN). CIN, caused by persistent chromosome segregation errors, leads to micronuclei formation and rupture, releasing genomic DNA into the cytosol and chronically activating the cGAS–STING pathway. Unlike acute DNA damage responses, CIN-driven STING activation preferentially triggers noncanonical NF-κB signaling rather than IRF3-dependent IFN-I responses, thereby promoting immune evasion and metastasis [104]. Single-cell transcriptomic analyses further reveal that CIN rewires downstream STING signaling, weakening IFN-I responses while enhancing ER stress pathways, which fosters an immunosuppressive and pro-metastatic tumor microenvironment. Functionally, reversing CIN, depleting tumor-intrinsic STING, or inhibiting ER stress signaling significantly suppresses metastasis, and STING inhibition reduces CIN-driven dissemination in melanoma, breast, and colorectal cancer models. Clinically, CIN-associated micronuclei, cGAS activation, and immunosuppression are prevalent in triple-negative breast cancer [105]. Taken together, chronic STING activation driven by CIN acts as a key driver of resistance and metastasis, highlighting the need for CIN-specific strategies to reprogram STING signaling and restore effective antitumor immunity.

Beyond CIN, other genetic and epigenetic alterations—such as loss-of-function mutations in STING or cGAS, promoter hypermethylation [106], or overexpression of DNA exonucleases like TREX1 [107]—may also diminish the efficacy of STING agonists. These alterations highlight the necessity of incorporating genomic and epigenomic profiling into patient stratification to identify tumors most likely to benefit from cGAS–STING–based therapies.

#### TME related barriers

The TME harbors a complex immunosuppressive network that significantly limits the antitumor efficacy of cGAS–STING activation. Tregs can secrete IL-10 and IDO to suppress antigen-specific CD8⁺ T cell activity, thereby weakening cytotoxic immune responses [103]. In addition, activation of the STING–IL-35 signaling axis in B cells markedly inhibits NK cell proliferation and effector function, further compromising innate immune defense [42]. These immunosuppressive mediators form a robust negative feedback loop that hinders the ability of exogenous cGAS–STING activation to achieve durable antitumor effects.

TAMs also play a dual role in cGAS–STING–mediated immune responses. On one hand, M1-like TAMs can be pharmacologically reprogrammed to enhance antitumor immunity. For example, a prodrug STING agonist (GB2) targeting TREM2⁺ TAMs promoted a shift toward an inflammatory M1 phenotype, increased phagocytic activity, and enhanced CD8⁺ T cell infiltration, ultimately leading to complete tumor regression in murine colorectal cancer models without systemic toxicity [108]. On the other hand, dysregulated interactions between tumor cells and TAMs may undermine this effect. In NSCLC, connexin 43 (Cx43)–mediated gap junction transfer of cGAMP from tumor cells to macrophages is required for optimal STING activation and T cell priming. Loss of Cx43 reduced T cell activation, promoted M2-like polarization, and conferred resistance to PD-1 blockade, thereby highlighting how impaired cGAS–STING signaling in TAMs can facilitate immune evasion [109]. Moreover, MDSCs can impair T cell metabolic activity through the generation of reactive oxygen species (ROS) and the release of arginase, reducing the persistence of antitumor immunity. Therefore, future therapeutic strategies should go beyond simply activating the cGAS–STING pathway. A rational approach may involve combining cGAS–STING agonists with immune checkpoint inhibitors, Treg or MDSC–depleting agents, and metabolic pathway modulators to dismantle TME-driven immunosuppressive barriers. Furthermore, the use of nanodelivery platforms or cell-targeted STING agonists to selectively activate immune effector cells within the tumor site could maximize antitumor efficacy while minimizing systemic toxicity risks.

Another emerging barrier is the role of stromal and endothelial cells in the TME, which can limit immune cell infiltration despite STING activation. Cancer-associated fibroblasts (CAFs), for example, may deposit dense extracellular matrix that physically restricts T cell trafficking [110, 111]. Addressing these stromal components—through matrix remodeling agents or targeted delivery systems—could further enhance the efficacy of cGAS–STING + ICI combination therapy.

#### Immune-Related toxicities

From a translational perspective, systemic administration of cGAS–STING agonists carries a risk of acute immune-related toxicities, including transient cytokine storm–like responses and flu-like symptoms [112]. Non-nucleotide small-molecule agonists may also induce T cell apoptosis due to the high STING expression in these cells [113]. Intravenous or intraperitoneal delivery can further overactivate the pathway in normal tissues, causing unwanted inflammation [114].

Importantly, ICIs such as anti–PD-1/PD-L1 and anti–CTLA-4 antibodies are also associated with a broad spectrum of immune-related adverse events (irAEs) due to excessive activation of the immune system. These toxicities can involve multiple organ systems, including dermatologic reactions, colitis, hepatitis, pneumonitis, endocrinopathies, and, in rare cases, severe myocarditis or neurotoxicity [115, 116]. Importantly, early-phase clinical trials have provided more detailed safety information. For example, in a phase Ib study of ADU-S100 (MIW815) plus spartalizumab (NCT03172936), the most common treatment-related AEs were pyrexia (22%), injection site pain (20%), and diarrhea (11%), and the maximum tolerated dose was not reached. Similarly, in the phase I ulevostinag (MK-1454) trial (NCT03010176), pyrexia occurred in up to 70% of patients, accompanied by increases in proinflammatory cytokines such as CXCL10, IFN-γ, and IL-6. In a subsequent phase II study in HNSCC (NCT04220866), pyrexia was also frequent (62.5%) alongside modest improvements in median progression-free survival (6.4 months). For non-CDN agonists, MK-2118 in a first-in-human phase I trial (NCT03249792) caused grade 3/4 treatment-related AEs in 22–23% of participants, though these events were generally manageable. Collectively, these results suggest that while STING agonists are generally well tolerated, fever and injection-site reactions are the most common toxicities, and systemic immune activation may lead to more serious but manageable irAEs. Combination regimens that integrate cGAS–STING agonists with ICIs may further amplify the incidence and severity of such toxicities by synergistically enhancing immune activation. Therefore, careful dose optimization, biomarker-guided patient selection, and early monitoring for irAEs remain critical to ensuring an optimal balance between efficacy and safety in clinical practice.

Future clinical development should prioritize tissue-targeted delivery platforms and selective modulators to maximize tumor-specific effects while minimizing systemic immune activation [117]. Advances in nanotechnology and synthetic biology may help mitigate toxicity risks. For instance, stimuli-responsive nanoparticles that release STING agonists only under hypoxic or acidic tumor conditions could reduce systemic exposure [118]. Similarly, engineered probiotics or viral vectors designed to locally deliver STING agonists represent innovative approaches to enhance safety profiles while maintaining therapeutic potency [119].

## Conclusion and prospect

The integration of cGAS–STING agonists with immune checkpoint inhibitors holds substantial promise for reshaping the landscape of cancer immunotherapy. Preclinical evidence consistently supports their synergistic potential, with early-phase clinical trials revealing manageable safety profiles and encouraging, albeit heterogeneous, efficacy signals across diverse tumor types. Nonetheless, the translation of this strategy into routine oncology practice is constrained by multifaceted challenges. Tumor-intrinsic resistance, exemplified by chromosomal instability–associated immune evasion, and the immunosuppressive tumor microenvironment—dominated by Tregs, myeloid-derived suppressor cells, and immunoregulatory B cells—can significantly diminish therapeutic benefit. Furthermore, systemic activation of the cGAS–STING pathway risks triggering acute immune-related toxicities, including cytokine storm–like responses and multi-organ irAEs, especially when combined with ICIs.

To maximize clinical impact, future research should prioritize biomarker-driven patient selection, enabling tailored application to tumors most likely to benefit from cGAS–STING activation. Promising biomarkers include: (i) genomic and epigenomic alterations such as cGAS or STING loss-of-function mutations, promoter hypermethylation, or TREX1 overexpression; (ii) TME composition, particularly the abundance of Tregs, MDSCs, and M2-like macrophages that restrict STING-mediated immunity; (iii) CIN status, as tumors with high CIN may exploit chronic cGAS–STING signaling for immune evasion; and (iv) pharmacodynamic indicators such as IFN-I, CXCL9, and CXCL10 induction following treatment. Incorporating genomic profiling, TME characterization, and cytokine response monitoring into clinical trial design could refine patient stratification, reduce unnecessary toxicity, and enhance therapeutic benefit.

Additional, rational combination regimens that simultaneously dismantle multiple immunosuppressive networks—through Treg depletion, MDSC modulation, metabolic pathway targeting, or angiogenesis blockade—may help overcome resistance and sustain immune activation. Advanced delivery platforms, including nanoparticle-based carriers, immune cell–directed agonists, and tumor-targeted prodrugs, could enhance intratumoral drug concentration while minimizing systemic exposure. Beyond conventional ICIs, integrating cGAS–STING–based interventions with emerging modalities such as CAR-T or TCR-engineered T cells, bispecific antibodies, and personalized neoantigen vaccines offers opportunities to achieve more comprehensive and durable antitumor immunity. With continued mechanistic insights, technological innovation, and rigorous clinical validation, cGAS–STING agonist–based combination therapy has the potential to evolve from a promising experimental approach into a cornerstone of precision immuno-oncology.

## Data Availability

No datasets were generated or analysed during the current study.
